# Structural and Physicochemical Properties of Starch from Rejected Chestnut: Hydrothermal and High-Pressure Processing Dependence

**DOI:** 10.3390/molecules28020700

**Published:** 2023-01-10

**Authors:** Enrique Pino-Hernández, Luiz Henrique Fasolin, Lina F. Ballesteros, Carlos A. Pinto, Jorge A. Saraiva, Luís Abrunhosa, José António Teixeira

**Affiliations:** 1 CEB—Centre of Biological Engineering, University of Minho, Campus de Gualtar, 4710-057 Braga, Portugal; 2 LABBELS—Associate Laboratory, 4710-057 Braga, Portugal; 3 INOV.LINEA—Agri-Food Technology Transfer and Valorization Center, TAGUSVALLEY—Science and Technology Park, 2200-062 Abrantes, Portugal; 4 Department of Food Engineering, School of Food Engineering, University of Campinas, Campinas 13083-862, SP, Brazil; 5 LAQV-REQUIMTE, Chemistry Department, University of Aveiro, Campus Universitário de Santiago, 3810-193 Aveiro, Portugal

**Keywords:** *Castanea sativa*, high-pressure processing, pasting, rejected fruits, novel processing, starch digestion

## Abstract

The quality standards for the export of chestnuts generate large quantities of rejected fruits, which require novel processing technologies for their safe industrial utilization. This study aimed to investigate the impact of high-pressure processing (HPP) and hydrothermal treatments (HT) on the physicochemical properties of rejected chestnut starch. Chestnuts were treated by HPP at 400, 500, and 600 MPa for 5 min and HT at 50 °C for 45 min. In general, all HPP treatments did not induce starch gelatinization, and their granules preserved the integrity and Maltese-cross. Moreover, starch granules’ size and resistant starch content increased with the intensity of pressure. Native and HT chestnut starches were the most susceptible to digestion. HPP treatments did not affect the C-type crystalline pattern of native starch, but the crystalline region was gradually modified to become amorphous. HPP-600 MPa treated starch showed modified pasting properties and exhibited the highest values of peak viscosity. This study demonstrates for the first time that after HPP-600 MPa treatment, a novel chestnut starch gel structure is obtained. Moreover, HPP treatments could increase the slow-digesting starch, which benefits the development of healthier products. HPP can be considered an interesting technology to obtain added-value starch from rejected chestnut fruits.

## 1. Introduction

Starch is a carbohydrate composed of two polysaccharides, amylose, and amylopectin, which are its principal components (≈99%) in dry weight. This plant-based ingredient is presented in the form of granules of different sizes, shapes, and diameters, depending on the botanical origin and source. In the European Union (EU), starch comes primarily from processing cereals and tubers such as wheat, maize, and potatoes [1]. Nonetheless, the isolation of starch from non-conventional sources such as sorghum, barley, pulses, seeds, and fruits has been gaining industrial relevance in numerous food and non-food applications. These starch sources have different physicochemical and functional properties due to the differences in morphology of starch granules, amylose to amylopectin ratio, and crystalline pattern which may fulfil the processing requirements [2].

The chestnut fruit is one of the EU’s most important tree nut crops. According to the official data [3], the average annual chestnut production in the EU reached 240 thousand tons between 2016 and 2020. Portugal is one of the three primary producers in the EU, having an average annual production of 35 thousand tons during that period. Nonetheless, unlike other edible nuts, chestnuts are fruits with relatively high moisture content and metabolic activity, which frequently compromise their shelf-life and accelerate their post-harvest decay, causing high losses. For this reason, fresh chestnuts need to be sanitized before their commercialization. The most common treatment used by the industry involves immersing chestnuts in hot water (47–50 °C) for several minutes (30–45 min) to remove floating fruits and guarantee the inactivation of spoiling microorganisms and insect larvae. Afterward, the chestnuts usually pass through a calibration process that ends with the rejection of small chestnuts, fruits with double embryos, or cracked shells. In this context, technologies that can exploit the rejected chestnuts and convert them into high-value-added products are worthy of investigation.

Chestnut kernel is rich in carbohydrates, namely in starch, which accounts for more than 60% of its dry weight [4], a value almost twice the starch content in potatoes [5]. Chestnut starch is mainly available as a by-product from rejected chestnuts due to the quality standards demanded by the fresh market. This product is gluten-free and consists of starch granules from C-type with relatively high crystallinity, composed of 26.5–56.9% amylose, 36.8–49.5% resistant starch, and 6.3–20.0% slowly digestible starch, depending on the chestnut variety [6,7,8,9]. Another characteristic of chestnut starch is its lower swelling capacity which leads to faster destruction of starch granules and, consequently, to lower gelatinization temperatures, low thermal stability, and high retrogradation [6,10]. Thus, native chestnut starch has some functional limitations for industrial processing [4,11].

Thermal treatments are conventionally used to physically modify starch by applying temperatures from 50 to 140 °C and processing times between 15 and 960 min. Under these treatment conditions, rice, chestnut, pea, lentil, cowpea, and Dioscorea starches and gels undergo irreversible gelatinization viscosity, strength, and hardness changes, and their crystal structure is lost without destroying the granular structure while modifying starches’ thermal and functional properties [5,11,12]. Therefore, the modification of chestnut starch using novel technologies that provide shorter processing times and reduced energy consumption can be a solution to improve its functionalities and structural characteristics and expand its use by different food industries that demand more sustainable processes.

Recently, several thermal and nonthermal innovative methods, such as infrared and microwave radiations [7], ultrasonic [13], and high-pressure processing (HPP) [9], have been explored to overcome the existing drawbacks of thermally modified chestnut starch and flour. However, there is limited information concerning the impact of HPP on the chestnut starch properties.

High-pressure processing (HPP) is a nonthermal processing technology that has been used to study the modification of native starch properties from different food matrices, such as lentil, pea, quinoa, maize, and potato, by applying pressures from 100 to 600 MPa and processing times from 2 to 30 min [14,15,16]. This method leaves no chemical residues and has the advantages of simplicity and safety compared with other methods of starch modification. Additionally, HPP technology can delay starch retrogradation, induce cold gelatinization, increase starch swelling without destroying starch granules, modify the pasting properties of treated starches, change their composition in slowly digestible and resistant starch, as well as some physical properties such as starch granule morphology, crystallinity degree, starch color, and gel texture.

In this context, this work aimed to study the impact of HPP at 400, 500, and 600 MPa on the starch from rejected chestnuts by evaluating their physical, chemical, thermal, mechanical, rheological, morphological, and structural properties.

## 2. Results and Discussion

### 2.1. Long- and Short-Range Molecular Order

**Figure 1:**
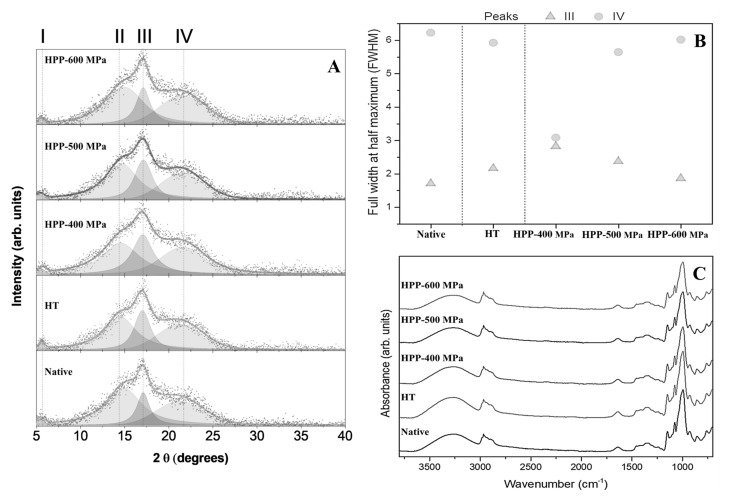
XRD patterns (**A**), peaks deconvolution plot (**B**), and FTIR-ATR spectrum (**C**) of chestnut starches.

The XRD patterns of the chestnut starches are presented in Figure 1A. Results revealed that the HPP treatments led to slight modifications in starches. The peaks (2θ = 5°, 15°, 17°, and 22°) identified for the chestnut starch samples (Figure 1A) indicate a typical C-type crystalline structure, which is a mixture of A and B type XRD patterns [9,14]. As shown in Figure 1B, the full width at half maximum (FWHM) values of peaks I and II of the starch samples (around 0.92 and 4.73, respectively) did not show any significant difference, while peaks III and IV (Figure 1A) showed a monotonic evolution in opposite directions as the HPP pressure increased. The diffraction peak at 17° (peak III, amylose XRD patterns) narrows as the pressure increase, whereas the diffraction peak at 22°–23° (peak IV, amylopectin XRD patterns) broadens. This behavior could be due to the fast decompression of the HPP vessel (less than 3 s) that occurred immediately after the treatments, as it may have caused alterations in the secondary and tertiary structures of starch granules (crystalline region, amylopectin). These results can be associated with morphological (SEM) and thermal (DSC) desirable changes in starch granules.

FTIR spectra of native and modified chestnut starches showed a characteristic carbohydrate pattern [13]. The absorption bands typical for chestnut starches are summarized in Table S1 (Supplementary Materials). FTIR spectra evidenced the same structure, and chemical bonds for all the starches analyzed, regardless of the sample processing (Figure 1C), suggesting that HT and HPP treatments only produced physical changes in the samples. This evidence can be related to the fact that the crystalline structure of the starch granules was not destroyed. This result agrees with the microscopical observations, where it was revealed that the Maltese-cross is visible and the structure of treated starch was not destroyed. Similar results about the absorption peaks characteristic were previously obtained in chestnut starch treated by ultrasound and microwave [13].

### 2.2. Rheological Measurements

**Figure 2:**
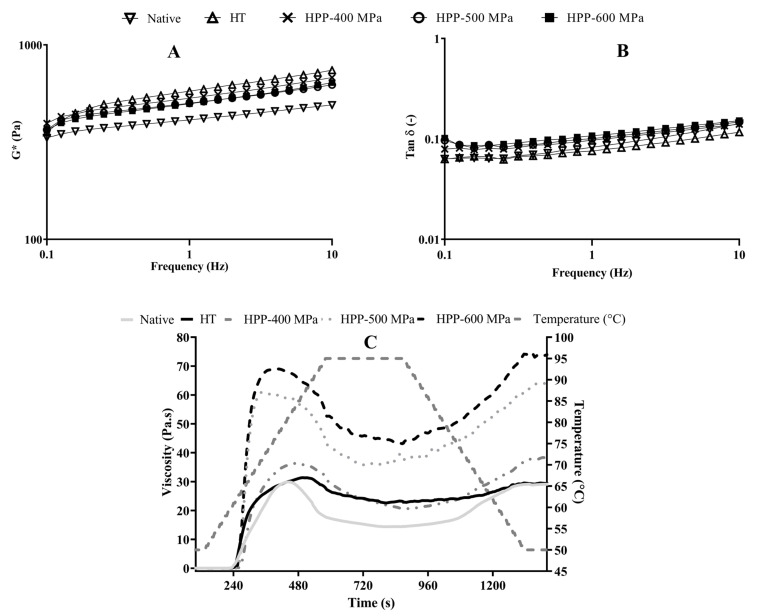
Complex modulus (G*) (**A**) and tan δ as a function of frequency (**B**) and pasting curves (**C**) of native and treated chestnut starch samples.

The frequency sweeps (Figure 2) allowed us to confirm the gel formation and evaluate its viscoelastic behavior at low shear. The analysis of G* showed a gel-like behavior with slight frequency dependence that is typical of soft gels (Figure 2A). All treated systems had higher G* values in comparison to native starch. However, the values for HT were slightly higher than for HPP samples, revealing a more structured system. Moreover, tan δ curves showed the prevailing elastic modulus, and their magnitude was just below 0.1, which is typical of a soft gel structure [17]. The HT and native gels, despite the proximity to the HPP samples (Figure 2B), presented lower tan δ values, that is, the predominance of G’. This denotes an increase in the elastic component, which is compatible with a more networked gel structure, mainly at low frequency. This characteristic could be related to the higher stability of the gel.

On the other hand, the pasting profiles of treated starch showed different behaviors when compared to each other and with the native starch (Figure 2C). The native starch showed the lowest values of the pasting properties (peak viscosity, trough viscosity, and final viscosity), while HPP-treated starch showed a lower pasting temperature than native and HT starches. The pasting temperature values agree with the temperature peak values obtained from DSC analysis (Table 1). Several authors reported similar behaviors when comparing HPP effects on native starch samples. Namely, the pasting temperature of lentil starch decreased by around 3% after HPP treatment [18] and by 11% in quinoa and 6.3% in maize starches when pressure levels increased from 0.1 to 500–600 MPa [19].

Regarding HPP treatments, the increase in pressure level led to additional modifications in starches’ pasting properties, setback, and breakdown viscosity (Table 1). The maximum viscosity reached during the pasting analysis is called the peak viscosity, which shows the ability of starch granules to absorb water and swell before gelatinization/breakdown [20].

HPP-600 MPa treated samples had the highest values of peak viscosity, trough viscosity, final viscosity, setback, and breakdown. The higher pressure of this treatment could force the entrance of free water into the starch granules since, as seen in the SEM images (see Section 2.4), they increased in volume. Thus, the friction between granules increased, and consequently, the samples’ viscosity increased too. Similar behavior in peak viscosity has been reported for HPP-treated pea starch, as it suffered almost no change up to 400 MPa but increased with pressures of 500 MPa and 600 MPa [21].

On the other hand, breakdown increased significantly when the pressure increased from 400 to 600 MPa, showing that 600 MPa-treated starch pastes are highly susceptible to disintegration during the heating process, probably due to the formation of weaker structures. This parameter can be used to identify the stability of starch gels against shearing and high temperatures after gelatinization [20]. The growing tendency of breakdown viscosity observed in HPP-treated samples was associated with higher peak viscosities and the modifications induced by HPP on the granules’ size (see Section 2.4). As observed by SEM, after the HPP treatments, the swelling degree increased significantly (see Section 2.4). The breakdown behavior obtained here (HPP—400 to 600 MPa for 5 min) is contrary to the decreasing tendency reported in [9] for chestnut samples treated by HPP from 0.1 to 600 MPa during 10 min. The difference between these behaviors can result from the longer processing times used and the loss of birefringence of the starch granules.

Regarding the setback, this parameter increased significantly when the pressure increased from 400 to 600 MPa, revealing that pressure-treated starch pastes had a higher retrogradation tendency [9,20]. Starch with a high retrogradation tendency is desirable in some food applications due to its role in modifying the final product structure, and the mechanical and sensorial properties, and because it increases SDS and RS [15].

### 2.3. Digestible and Resistant Starch Contents

**Figure 3:**
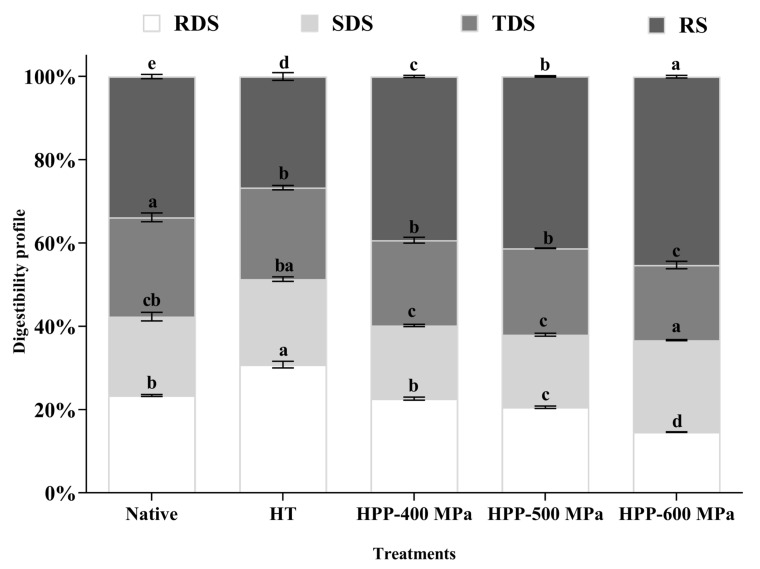
Content of RDS, SDS, TDS, and RS of native and treated chestnut starch samples. Error bars show SD. Bars with different letters indicate a significant difference among treatments means within each starch digestion fraction (*p* < 0.05).

Starch fractions, RDS, SDS, TDS, and RS, of native and treated chestnut starches samples are shown in Figure 3. Native and HT chestnut starches were more susceptible to digestion than HPP-treated starches, registering higher RDS and TDS and lower RS contents. However, the SDS content of native starch was significantly higher than HPP-treated starches. The higher susceptibility of native and HT starch to enzymes could be attributed to its smaller granule size, lower peak viscosity, and ∆H. The HT treatment may have potentiated the disruption of hydrogen bonds existing on starch chains, promoting the granules swelling and rupture and the access of digestive enzymes to starch chains [22]. On the other hand, the native starch digestion values obtained in this study showed similar relative values to those obtained for other chestnut starches (RDS: 18.0%; SDS: 43.0%; RS: 39.0%) [22].

As shown in Figure 3, regardless of the pressure level used in the HPP-treated starches, the digestibility degree was reduced compared with native starch, registering a decrease in RSD and an increase in SDS and RS contents. This behavior may be associated with starch granule size and structure and their lower susceptibility to enzymatic hydrolysis after treatments [23]. A similar starch digestion behavior has been reported in common buckwheat starch treated by HPP [23].

The increase of RS after HPP at 600 MPa (Figure 3) was also previously found in lentil, aquafaba, and Tartary buckwheat samples [18,24,25]. HPP could increase RS content due to strong amylose-amylose and amylose-amylopectin interactions formed after the treatment [15,23]. Moreover, this behavior can be related to the adiabatic effect resulting from the pressurization/depressurization of the high-pressure vessel where the samples are confined. This effect can induce physical and chemical changes, such as starch nuclei formation and starch recrystallization, leading to increases in resistant starch [14,25]. From a practical point of view, starch treated by HPP-600 MPa can potentially be used on food products with added health benefits since their higher content in SDS and RS have a lower starch hydrolysis degree. These properties can be relevant to the glycemic index and the prevention of non-insulin-dependent diabetes, as well as positively modulate the gut microbiota, functioning as a prebiotic.

### 2.4. Morphological Structure

**Figure 4:**
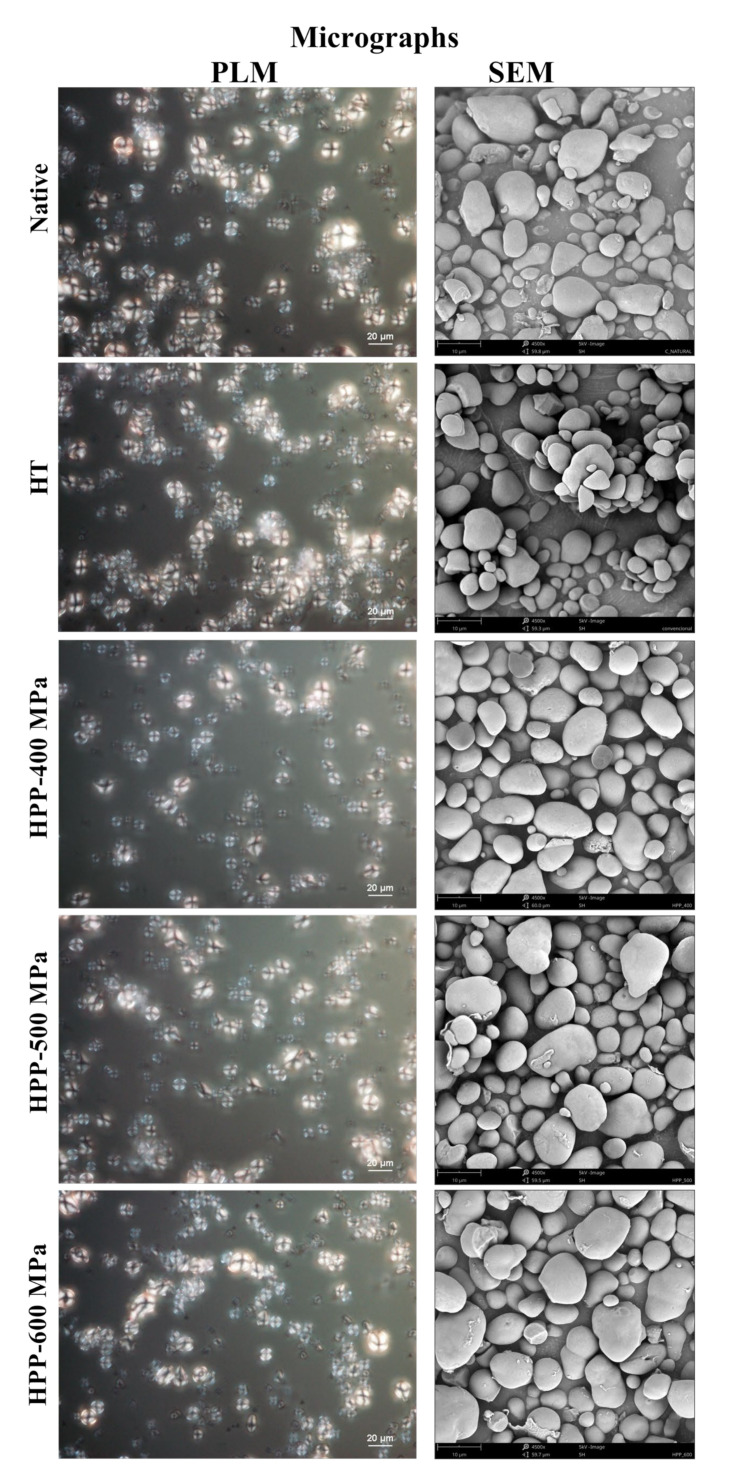
PLM and SEM micrographs of chestnut starch granules samples.

The changes in birefringence of native and treated starch are shown in Figure 4. All the starch samples showed bright and regular Maltese crosses, typical of ungelatinized starch granules. Thus, the birefringence of the native and treated starches was not affected by the temperature and time used during HT, nor by the pressure level used in HPP. These results can be attributed to the fact that the chestnut starch has intermediate amylose content (30%) in the granule. The polarized light results of HPP-treated starches obtained in this study are according to results previously reported in [26] for HPP (>600 MPa) treated corn, waxy corn, wheat, and potato starches, as no differences in birefringence compared to native starch were also found.

On the other hand, no significant modifications were also observed in the chemical characterization of the starch samples, as the X-ray spectrometer (EDS) test gives average carbon and oxygen contents of 74.6 ± 1.0% and 25.4 ± 1.8%, respectively. Relatively to morphological changes of starch granules induced by the processing technologies, modifications after HT and HPP treatments were not found. Nonetheless, the results revealed significant differences in size and volume-surface diameter (d_32_) between native and treated starch granules.

The SEM images of native and treated chestnut starch granules are shown in Figure 4. The surface of native starches granules appeared smooth, showing no evidence of fissures or cracks, and having round or oval shapes. The starch granules diameter (d_32_) of the native samples registered a value of 5.9 ± 0.3 μm, with 80% of the population having 1.5 to 6.0 μm (frequency histograms in Figure S1). HPP treatments considerably influenced the starch granules’ size as the pressure level increased from 400 to 600 MPa (Figure 4). The largest starch granules were registered on the HPP-600 MPa samples, with 70% of the population having particles between 4.5 and 10.5 μm. This increase in size could explain the changes in pasting properties (temperature and peak viscosity) described in Section 2.2. Overall, the pressure levels and processing time used in this study did not lead to starch granules’ structural disruption and gelatinization, which agrees with DSC peak temperature results (Table 1). Similar results were previously obtained for potato starch granules processed at 400 MPa, as no granule destruction was observed [15]. Moreover, in [9] it was found that chestnut starch treatment with pressures between 400 to 600 MPa for 10 min increased starch swelling without changing the starch granules morphology. However, in [27] it was found that rice starch granule size increased significantly with increased pressure levels, from 120 to 600 MPa for 30 min, with an irreversible loss of the particle structure at 600 MPa. In this case, the higher processing times may have been the influencing variable.

### 2.5. Thermal Properties

Thermal transition parameters showed significant differences in some parameters among the different treatments (Table 2). DSC thermograms revealed for all treatments only one event that showed a phase transition with an endothermic melting/gelatinization peak. These results agree with the XRD and microscopy results (Figure 1 and Figure 4). The parameter values T_o_, T_p_, T_c_, and ∆H obtained in this study for the native starch are similar to those (T_o_ 58.5 °C, T_p_ 64.0 °C, T_c_ 68.9 °C, and ∆H 3.4 J/g) previously reported also for chestnut starch [28]. On the other hand, the lowest ∆H value was obtained for the HT samples, suggesting that a higher gelatinization of starch (around 13%) has been induced in this treatment. This behavior could result from the significant modification of amylopectin structure due to the prolonged heating time. A significant reduction in ∆H value was also previously observed by [28] when comparing ∆H values of heat-treated chestnut starch (0.6 J/g) to untreated samples (3.4 J/g).

In the HPP treatments, both onset and gelatinization peak temperature values showed a decrease of about 1.3% and 1.0%, respectively compared to the native starch sample. Regarding the enthalpies (ΔH), the HPP and native starch samples presented similar values. However, the gelatinization peaks obtained in this study were lower than the obtained (68.0 °C) in [9] for HPP-treated chestnut flours (400 and 500 MPa for 10 min), even if the ΔH value (4 J/g) is similar to our data. Additionally, lentil starch treated by HPP at 400 MPa for 10 min retained the enthalpy of gelatinization constant [18]. Still, the authors observed a decrease of ΔH and gelatinization peak values with 500 MPa and no gelatinization peak with 600 MPa, suggesting that partial and complete gelatinization of starch occurred in those conditions, respectively. Therefore, the gelatinization process of high-pressure treated starch can be influenced by HPP process conditions, starch source, and amylose and amylopectin contents.

The TGA parameters showed significant differences in all treatments, except for the mass loss at the second decomposition stage (Table 1). The thermal decomposition of the native and treated starches occurred in three main degradation steps (mass loss) due to the phase transitions observed after heating the samples until 500 °C. This result agrees with [29], who also observed that chestnut oligosaccharides degradation occurred in three steps. The first degradation step for native and HT samples, corresponding to samples’ dehydration and loss of volatile components, occurred approximately at 71.0 °C with mass losses of approx. 9.3% and 11.5%, respectively. Regarding the HPP samples, the mass loss remained unchanged compared to native samples independently of pressure level, but the onset temperature increased around 6.9% in 500 and 600 MPa samples. It could be associated with starch granules’ higher resistance to heat treatment. This first step was also reported for oligosaccharides obtained from chestnuts [29].

The most substantial mass losses occurred during the second step at approximately 277–298 °C, reaching a maximum decomposition temperature of 317.5 °C. This step could be attributed to the depolymerization and decomposition of starch, as it produced mass losses of around 65.0% in all treatments. These changes may result from some physicochemical modifications induced by the HPP treatments in starch samples. Nonetheless, these results also provide evidence that native and treated starches could withstand high process temperatures, suggesting their versatility to be used in the food industry. Finally, the third thermal step started at approximately 450 °C, with around 24.5% of the native and treated starch samples remaining undegraded as a charred residue. Similarly, the char residue of chestnut samples when heated over 400 °C was 30.9% in [29].

### 2.6. Mechanical Properties of Starch Gels

**Figure 5:**
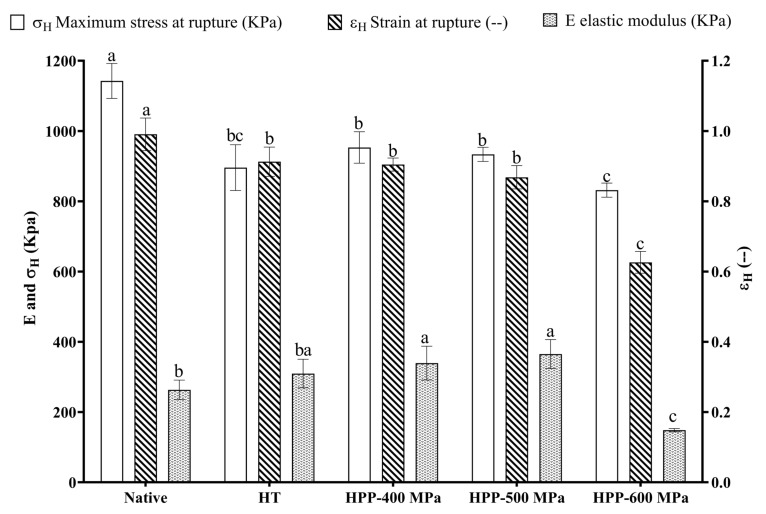
Mechanical properties of native and treated chestnut starch gel samples. Error bars show SD. Bars with different letters indicate a significant difference among treatments means within each mechanical property (*p* < 0.05).

The typical stress-strain curves obtained from the uniaxial compression measurements (Figure S2) show a unique tendency for all samples and describe starch gels with a clear rupture point. The mechanical properties of chestnut starch gels are shown in Figure 5. The native starch gel samples showed the highest hardness values (maximum stress at rupture—σH) and deformability (strain at rupture—εH). They also presented a stronger and more flexible gel structure as they break at higher stress and strain. In addition, their σH, εH, and elastic modulus E (kPa) values obtained in this study were higher than those (σH 4.1, εH 0.5, and E 2.8 kPa) previously reported, also for chestnut native starch [30]. These characteristics can be advantageous for gelling or thickening applications.

Relatively to the changes induced by the processing technologies, a significant decrease was observed in stress and strain at rupture after HT and HPP treatments (Figure 5). After the HT treatment, the modulus of elasticity did not differ significantly from native starch samples. However, with HPP treatments, differences were observed. In particular, starch gels from HPP-500 MPa showed a more elastic structure once the elastic modulus presented a higher value. On the other hand, HPP-600 MPa starch gel samples displayed a weaker and rapidly brittle structure as they broke at lower stress, strain, and elastic modulus. These modifications can benefit innovative applications involving precooked chestnut products where softening is desirable. These results are consistent with the increased starch granule size observed after the HPP treatments. Additionally, it can be related to the breakdown and final viscosity values obtained from pasting analysis (Table 1). Final viscosity indicates the resistance of starch to shearing and its ability of starch to form a gel [20]. In addition, similar results were obtained previously with chestnut flour [9], as they obtained weaker gels with increasing pressures (from 400 to 600 MPa). Thus, the physicochemical properties of starch, such as its crystalline/amorphous status and granules’ size, play an essential role in starch gels’ mechanical properties and can be used to modify the texture of food products [15,21].

## 3. Materials and Methods

### 3.1. Starch Non-Conventional Source

The rejected chestnut fruits of the variety “Martaínha” (*Castanea sativa* Mill.) were obtained from an industrial unit in Portugal. Chestnuts were packed in net bags by the wholesaler and transported fresh to the laboratory, where they were immediately prepared for analysis.

### 3.2. Treatments

Chestnut fruits were divided into batches to be treated differently using previously described processing conditions [31]. Batch 1 were untreated, and samples were named native starch. Batch 2 were hydrothermally treated at atmospheric pressure by dipping the fruits in water at 50 °C for 45 min with constant stirring and named HT. Batch 3 were vacuum-sealed and treated by HPP at 400, 500, and 600 MPa for 5 min at room temperature (20 °C). HPP-treated samples were named as follows: HPP-400 MPa, HPP-500 MPa, and HPP-600 MPa.

The HPP treatments were conducted in a 55 L high-pressure pilot unit (Hiperbaric 55, Hiperbaric S.A, Burgos, Spain). Water was used to pressurize samples at a rate of 3 MPa s^−1^, while decompression was performed in less than 3 s. Cold pressurizing water was used to maintain the temperature conditions during HPP treatment at room temperature (20 °C).

Before and immediately after each treatment, two kg of chestnuts was randomly taken from each batch. Next, the chestnut fruits were peeled, the inner skin was removed, and the peeled kernels were cut into small pieces and ground, frozen at −80 °C for 24 h (Thermo Scientific, Forma 8600 Series, Waltham, MA, USA), and freeze-dried at −50 °C in a vacuum chamber for 48 h (Christ, Apha 1–4 LSCplus, Osterode, Germany). Then, the freeze-dried samples were placed in a desiccator containing silica gel (~0% RH) for 6 h. Finally, the freeze-dried chestnuts were pulverized using a grinder (Taurus, Aromatic II, Lisbon, Portugal) and were sifted to obtain a 40-mesh powder.

### 3.3. Starch Isolation

Starch was isolated from chestnut flours by the aqueous solution method described previously [32] with some modifications. First, flours were obtained from freeze-dried chestnut kernels that were untreated or treated as described previously (Section 3.2), using the procedures previously reported [31]. Then, a slurry of 10% starch in distilled water was prepared and steeped for 12 h at room temperature. Next, the slurry was filtered through a 100-mesh sieve and centrifuged at 2500× *g* for 20 min at room temperature. After that, the supernatant was discarded, and the pellets were resuspended in 200 mL of distilled water. Finally, the slurry was again centrifuged, and the grey upper layer (containing fibers and proteins) was removed manually with a spatula. This last step was repeated 4 times to guarantee the total elimination of the grey layer.

Finally, the starch was resuspended in 100 mL of ethanol at 99.5% (*v*/*v*), filtered with a Whatman N° 5 filter paper, and dried in an oven (Thermo Electron, T6 Heraeus, Langenselbold, Germany) at 40 ± 0.2 °C for 15 h. The starch samples were ground using a grinder (Taurus, Aromatic II, Lisbon, Portugal), screened through a 100-mesh sieve, and stored at room temperature in sealed and dried polyethylene bags for further analysis. The native starch and HT starch samples were analyzed and compared with samples subjected to HPP treatments.

### 3.4. X-ray Diffraction

Crystalline phases of starch samples were evaluated by X-ray diffraction (XRD) using a D8 Discover diffractometer (Bruker, corporation, Billerica, MA, USA) with Cu tube (λ = 1.5406 Å). The radiation was generated at 40 mA and 40 kV, as previously reported [33]. The scattering angle of 2θ from 5° to 50° was measured at the step size of 0.04° and 1 s exposure at each step. On the other hand, to better understand the evolution of the crystallinity, a deconvolution of the peaks was carried out through Voigt functions, using the major peaks previously reported for the chestnut starch [34,35]. This method permitted defining the FWHM of the chestnut starch peaks.

### 3.5. Fourier-Transform Infrared Spectroscopy

The chemical groups and bonding arrangement of constituents were determined by Fourier-transform infrared spectroscopy (FTIR) using a Bruker Alpha II FTIR spectrometer equipped with a diamond attenuated total internal reflectance (ATR). The measurements were recorded over 4000–400 cm^−1^ at a resolution of 4 cm^−1^ and 24 scans per sample, as described [33]. This analysis was performed through Opus software (v. 7.0).

### 3.6. Rheological Measurements

Pasting measurements were performed in an HR-1 rheometer (TA Instruments, New Castle, DE, USA) equipped with a Peltier plate (40 mm, 1000 µm gap) as described by Gómez et al. [36]. Five grams of chestnut starch in 100 mL of distilled water with 24 h of hydration were used in the analysis. The heating ramp was set at 6 °C/min from 50 to 95 °C, and the cooling sweep was carried out at 2 °C/min from 50 °C to 6 °C.

The viscoelastic properties were evaluated by dynamic oscillatory measurements. Non-isothermal and frequency sweep analyses were carried out within the linear viscoelastic domain obtained from strain sweep (1% deformation—data not shown). The slurries were submitted to the same conditions used to evaluate the pasting behavior. Frequency sweeps were evaluated between 0.1 and 10 Hz. The elastic moduli (G’), viscous moduli (G”), complex moduli (G*) and tan δ were estimated.

### 3.7. Thermal Analyses

Differential Scanning Calorimeter (DSC) was conducted in an equipment DSC 6000 (Perkin Elmer, Waltham, MA, USA), and thermogravimetric analyses (TGA) were performed with an equipment TGA 4000 (Perkin Elmer, Waltham, MA, USA) as described previously [36].

The degree of gelatinization (% G) was calculated using the following Equation (1), previously reported in [21].

(1) $$\%\;G=\left(\frac{\Delta H_{cs}-\Delta H_{ps}}{\Delta H_{cs}}\right)\times \;100$$

where Δ*H_cs_* and Δ*H_ps_* are the gelatinization enthalpies of native starch and pressurized starch, respectively.

### 3.8. Morphological Properties and Size of Chestnut Starch Granules

Starch dispersions (50 mg/mL) in distilled water were prepared. One drop sample (25 μL) was taken for microstructural analysis by polarized light microscope (PLM). For observing starch granules’ birefringence in the form of Maltese crosses, micrographs were captured at room temperature with a Nikon Instruments Wide-Field Upright Ni-E Microscope (Tokyo, Japan) equipped with a digital camera using a 50× objective, a polarizer, and a NIS-Elements Microscope Imaging Software. In addition, micrographs and the elemental composition of the starches were obtained using a desktop scanning electron microscope (SEM) coupled to a dispersive energy X-ray spectrometer (EDS) (Phenom-World BV, Eindhoven, The Netherlands) as described previously [36].

The diameter and size of starch granules analysis were performed by measuring at least 300 granules in SEM micrographs using ImageJ software (v.1.46r for Windows). Measurements were used to calculate the mean diameter and particle size distribution to show frequency histograms. The average size was calculated considering the specific surface per unit volume (d_32_) described in Equation (2).

(2) $$d_{32}=\frac{\sum_{i}n_{i}d_{i}^{3}}{\sum_{i}n_{i}d_{i}^{2}}$$

where *n_i_* is the number of particles with diameter *d_i_*.

### 3.9. In Vitro Digestibility of Starch

The simulated in vitro chestnut starch digestion assay was carried out using the digestible and resistant starch assay kit (K-DSTRS—Megazyme), as previously described [37]. Rapidly digestible starch (RDS), slowly digestible starch (SDS), and total digestible starch (TDS) were determined at 20, 120, and 240 min, respectively. After 240 min of enzymatic digestion, the resistant starch (RS) was quantified. Absorbance was measured at 510 nm against blank using a UV/VIS spectrophotometer (Synergy HT, BioTek Instruments, Inc., Winooski, VT, USA).

### 3.10. Mechanical Properties of Starch Gels

Starch gels prepared by gelatinization of a 10% starch suspension in water at 95 °C for 30 min and stored for 24 h at 4 °C were used for mechanical analysis by uniaxial compression according to the method described previously [38] with some modifications. The analysis was performed at cold temperature using a Texture Analyzer (TA. HD plusC, Stable Micro Systems, Godalming, UK) equipped with a 5 kg load cell and a 60 mm diameter stainless-steel cylindrical probe. Starch gels cylinders (15 mm in length and diameter) were compressed at 1 mm/s up to 80% of their height and subsequently decompressed at 10 mm/s to their initial position. Assays were repeated ten times using ten different gels. The Hencky stress (σH) and strain (εH) were calculated from the force-deformation data according to [38].

### 3.11. Statistical Analysis

The results were compared by analysis of variance (ANOVA) and Tukey’s test at a significance level of 5% using GraphPad Prism v6. Ink software. The data reported are expressed as the average of triplicate observations ± SD.

## 4. Conclusions

HPP can be a helpful method to induce desirable modifications on the structural, granule size, pasting, thermal, mechanical, and color characteristics of chestnut starch. The HPP process is considered safe since it does not form toxic by-products. HPP-600 MPa treated starch can be used in products that require higher pasting properties and swelling power, setback, and breakdown viscosity, as well as gel structures with weaker and rapidly brittle tendencies. Nonetheless, HPP treatments used did not induce starch gelatinization. Chestnut starches may be used as an ingredient for food applications that require industrial processing at intermediate temperatures, such as those used in sterilization, pasteurization, and cooking methods. From a technological point of view, it was demonstrated here that HPP could be used to change chestnut starch characteristics and obtain ingredients with modified thermal and rheological properties that the food industry can use to develop products with novel textures and structures.

## Figures and Tables

**Table 1:** Pasting results of native and treated chestnut starch samples.

Parameters	Samples				
	Native	HT	HPP (MPa)		
			400	500	600
Pasting temperature (°C)	66.0 ± 0.3 ^a^	66.8 ± 0.2 ^a^	63.9 ± 0.0 ^b^	62.7 ± 0.2 ^b^	62.7 ± 0.3 ^b^
Gelatinization time (s)	290.8 ± 0.7 ^a^	298.5 ± 0.2 ^a^	272.5 ± 2.4 ^b^	257.7 ± 2.0 ^c^	257.5 ± 2.5 ^c^
Peak viscosity (Pa·s)	30.7 ± 0.6 ^d^	31.8 ± 1.0 ^d^	36.3 ± 2.1 ^c^	62.6 ± 1.7 ^b^	70.5 ± 1.5 ^a^
Peak time (s)	494.0 ± 4.2 ^b^	552.7 ± 7.5 ^a^	480.0 ± 2.0 ^c^	340.0 ± 5.0 ^e^	372.5 ± 4.5 ^d^
Trough viscosity (Pa·s)	14.3 ± 0.3 ^d^	23.1 ± 0.7 ^c^	20.6 ± 2.4 ^c^	35.2 ± 0.5 ^b^	42.7 ± 0.4 ^a^
Final viscosity (Pa·s)	26.8 ± 1.6 ^e^	30.5 ± 1.5 ^d^	40.5 ± 3.9 ^c^	65.2 ± 0.6 ^b^	74.0 ± 1.0 ^a^
Setback (Pa·s)	12.4 ± 1.4 ^c^	7.4 ± 1.6 ^d^	19.9 ± 0.5 ^b^	30.0 ± 2.0 ^a^	31.3 ± 1.4 ^a^
Breakdown viscosity (Pa·s)	16.4 ± 0.6 ^b^	9.0 ± 0.3 ^c^	15.7 ± 0.3 ^b^	27.4 ± 2.2 ^a^	27.8 ± 1.1 ^a^

Values are means ± standard deviation (SD). Different superscript letters on the same row indicate a significant difference at *p* < 0.05.

**Table 2:** Thermal properties of native and treated chestnut starch samples.

Parameters		Samples				
		Native	HT	HPP (MPa)		
				400	500	600
DSC	T_o_ (℃)	57.5 ± 0.1 ^a^	57.9 ± 0.2 ^a^	56.2 ± 0.0 ^b^	56.2 ± 0.0 ^b^	56.2 ± 0.1 ^b^
	T_p_ (℃)	61.6 ± 0.1 ^b^	62.4 ± 0.3 ^a^	60.4 ± 0.1 ^c^	60.8 ± 0.1 ^c^	60.8 ± 0.1 ^c^
	T_c_ (℃)	66.7 ± 0.1 ^a^	67.0 ± 0.3 ^a^	66.1 ± 0.1 ^a^	66.4 ± 0.1 ^a^	66.3 ± 0.2 ^a^
	∆H_gel_ (J/g)	3.8 ± 0.1 ^a^	3.2 ± 0.1 ^b^	4.1 ± 0.1 ^a^	3.9 ± 0.1 ^a^	3.7 ± 0.1 ^a^
TGA	T_o_ (℃)	72.2 ± 0.0 ^b,c^	69.8 ± 1.3 ^c^	72.0 ± 1.3 ^b,c^	78.3 ± 1.7 ^a,b^	79.9 ± 1.1 ^a^
	MLFDS (%)	9.3 ± 0.1 ^b^	11.5 ± 0.2 ^a^	9.6 ± 0.0 ^b^	9.4 ± 0.3 ^b^	8.9 ± 0.2 ^b^
	T_1_ (℃)	298.5 ± 0.7 ^a^	296.8 ± 0.6 ^a^	277.0 ± 0.7 ^b^	280.2 ± 0.4 ^b^	277.8 ± 0.0 ^b^
	T_d_ (℃)	315.1 ± 0.5 ^a^	314.0 ± 1.0 ^a^	317.6 ± 0.1 ^a^	315.2 ± 0.2 ^a^	315.0 ± 0.2 ^a^
	MLSDS (%)	64.5 ± 0.0 ^a^	64.8 ± 1.6 ^a^	64.0 ± 0.9 ^a^	65.5 ± 0.6 ^a^	66.3 ± 0.3 ^a^

T_o_, ‘onset’ temperature of weight losses; T_p_, gelatinization peak; T_c_, ‘endset’ or final temperature; ∆H_gel_, gelatinization enthalpy; MLFDS, mass loss at the first decomposition stage; T_1_, extrapolated temperature of decomposition; T_d_, temperature of decomposition; MLSDS, mass loss at the second decomposition stage. Values followed by different superscript letters on the same row indicate a significant difference between them at *p* < 0.05.

## Data Availability

Not applicable.

## Supplementary Materials

The following supporting information can be downloaded at: https://www.mdpi.com/article/10.3390/molecules28020700/s1, Figure S1: frequency histograms of native and treated chestnut starch samples. Figure S2: stress-strain curves of native and treated chestnut starch gel samples. Table S1: IR assignments of the main vibrations in the FTIR spectra from chestnut starch samples.

## Sample Availability

Samples of the compounds are not available from the authors.
