# Supplementary material for: Structural and Physicochemical Properties of Starch from Rejected Chestnut: Hydrothermal and High-Pressure Processing Dependence
Source: Molecules. 2023 Jan 10;28(2):700. doi: 10.3390/molecules28020700 (PMC9865283; doi:10.3390/molecules28020700)
Supplement: Supplementary file 1 [file molecules-28-00700-s001.zip › molecules-2101821-supplementary.pdf]

Frequency histograms

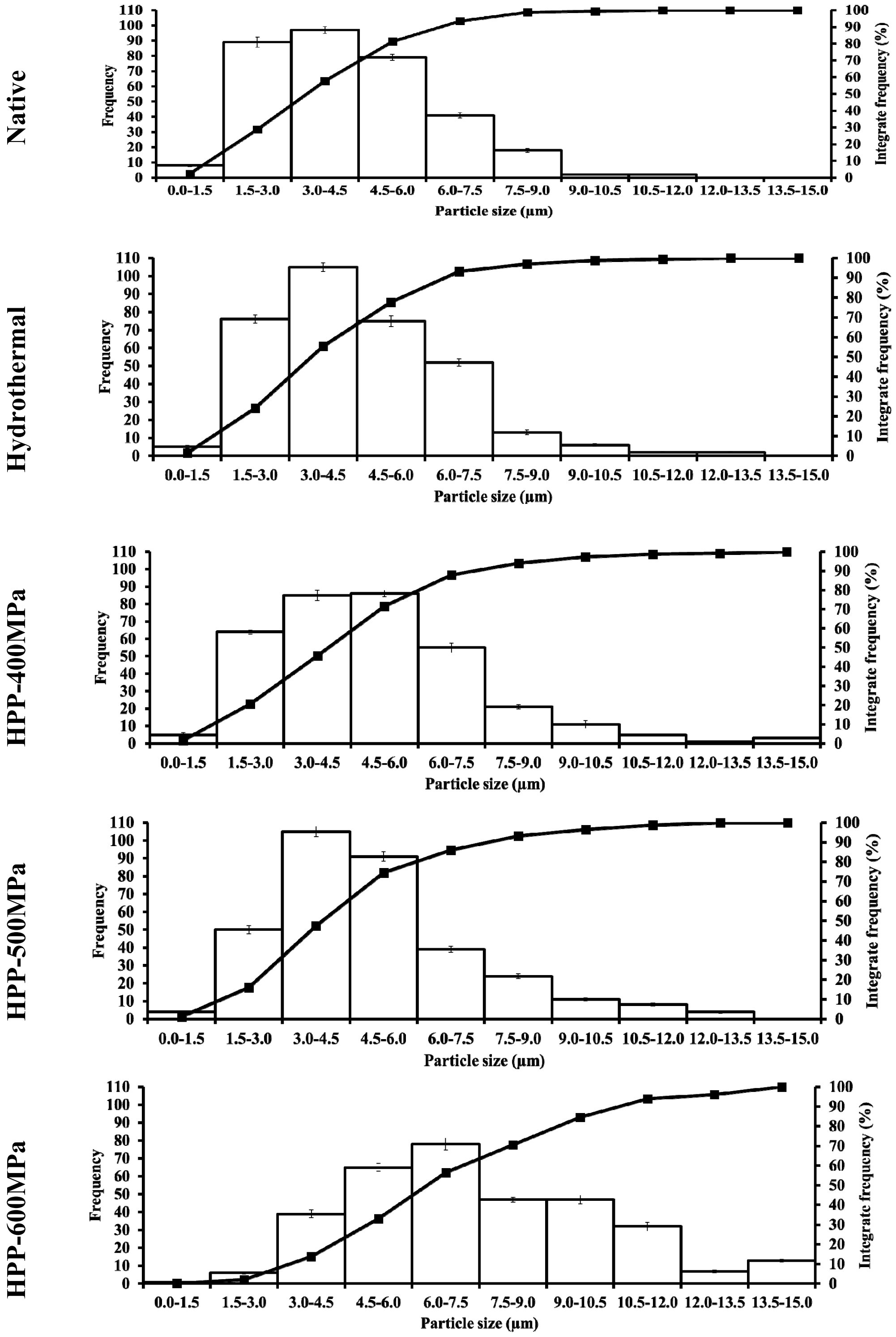

Figure S1: Frequency histograms of native and treated chestnut starch samples. Native – Control, Hydrothermal – Conventional, HPP – High-pressure processing.

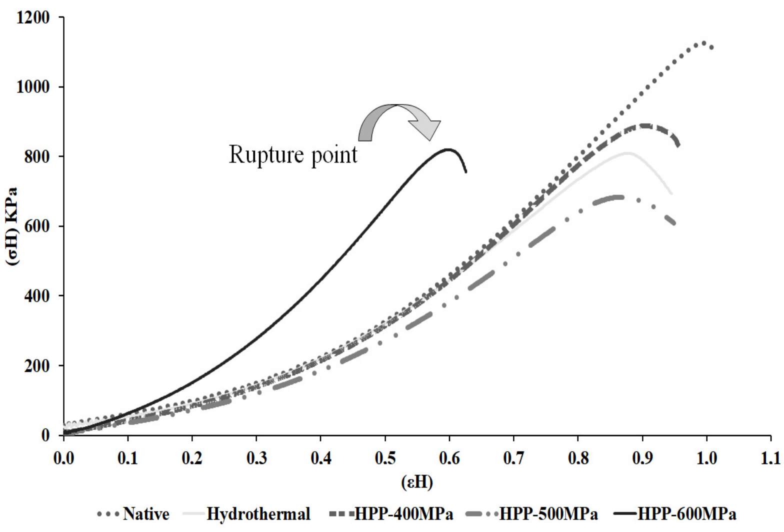

Figure S2: Stress-strain curves of native and treated chestnuts starch gel samples. Native – Control, Hydrothermal – Conventional, HPP – High-pressure processing.

Table S1: IR assignments of the main vibrations in the FTIR spectra from chestnut starch samples

Band frequency (cm^{-1})	Vibrations (cm^{-1}) Starch	Assignments
3600 - 3000	3273	O–H stretching broad band
3000 - 2800	2966	C–H asymmetric stretching band from CH_3 groups
	2925	C–H asymmetric stretching band from CH_2 groups
	2900	C–H symmetric stretching bands
	2877	
	2850	
1750 - 1500	1642	C=O stretching band from carboxylic acid group
	1455	C=C stretching bands from aromatic rings
1500 - 1200	1365	
	1335	C–O stretching bands
	1242	
	1147	
1200 - 650	1077	is related to bending vibrational modes of C–O existing in the pyranose form
	1039	C–O stretching bands (anhydro-glucose ring)
	1017	
	996	C–O bond from C–O–C in the starch
	928	C–O stretching bands (anhydro-glucose ring)
	860	C–O stretching bands
